# Exosome-mediated repair of spinal cord injury: a promising therapeutic strategy

**DOI:** 10.1186/s13287-023-03614-y

**Published:** 2024-01-02

**Authors:** Tong Yu, Li-Li Yang, Ying Zhou, Min-Fei Wu, Jian-Hang Jiao

**Affiliations:** 1 Department of Orthopedic, The Second Norman Bethune Hospital of Jilin University, Changchun, 130000 Jilin Province China; 2 Department of Operating Room, The Third Hospital of Qinhuangdao, Qinhuangdao, 066000 Hebei Province China

**Keywords:** Spinal cord injury, Exosome, Repair, Mechanism

## Abstract

Spinal cord injury (SCI) is a catastrophic injury to the central nervous system (CNS) that can lead to sensory and motor dysfunction, which seriously affects patients' quality of life and imposes a major economic burden on society. The pathological process of SCI is divided into primary and secondary injury, and secondary injury is a cascade of amplified responses triggered by the primary injury. Due to the complexity of the pathological mechanisms of SCI, there is no clear and effective treatment strategy in clinical practice. Exosomes, which are extracellular vesicles of endoplasmic origin with a diameter of 30–150 nm, play a critical role in intercellular communication and have become an ideal vehicle for drug delivery. A growing body of evidence suggests that exosomes have great potential for repairing SCI. In this review, we introduce exosome preparation, functions, and administration routes. In addition, we summarize the effect and mechanism by which various exosomes repair SCI and review the efficacy of exosomes in combination with other strategies to repair SCI. Finally, the challenges and prospects of the use of exosomes to repair SCI are described.

## Introduction

SCI is a catastrophic injury to the CNS that can lead to sensory and motor dysfunction and is characterized by a high rate of disability and mortality [1, 2]. Approximately 10.4–83 per 1 million people suffer from SCI each year worldwide. Furthermore, the morbidity and mortality rates are increasing each year. SCI still imposes significant costs on society and presents several challenges to the medical community [3]. After SCI, a series of pathophysiological changes occur at the lesion site, including inflammatory response and neuronal apoptosis, which are followed by the formation of cavities and scars, resulting in the inhibition of axonal regeneration [4]. Unfortunately, regeneration after SCI is extremely weak due to the low plasticity of the CNS and limited neuronal regeneration. Currently, there is no effective method to completely repair the function of the spinal cord after SCI [5].

Exosomes, which are extracellular vesicles of endoplasmic origin with a diameters of 30–150 nm, are secreted by cells [6]. Exosomes contains various biologically active molecules, including nucleic acids and proteins, which play a crucial role in intercellular communication [7, 8]. Importantly, exosomes show good stability, biocompatibility, biological barrier permeability, and low immunogenicity, which supports their use in the repair of tissue damage [9–11]. Furthermore, exosomes can carry new functional proteins through genetically engineered cells [12, 13]. In addition, exosomes can also be used as carriers of small molecules or nucleic acids to target drugs to specific types of cells or tissues [14–16]. Thus, exosomes are not only a potential tool for exploring the mechanism of SCI but may also be an important substance for repairing SCI in the future [17–19]. Consequently, in this article, we describe the preparation, functions, administration routes mechanism, and challenges of various exosomes in the treatment of SCI.

## Preparation of exosomes

### Isolation

**Fig. 1:**
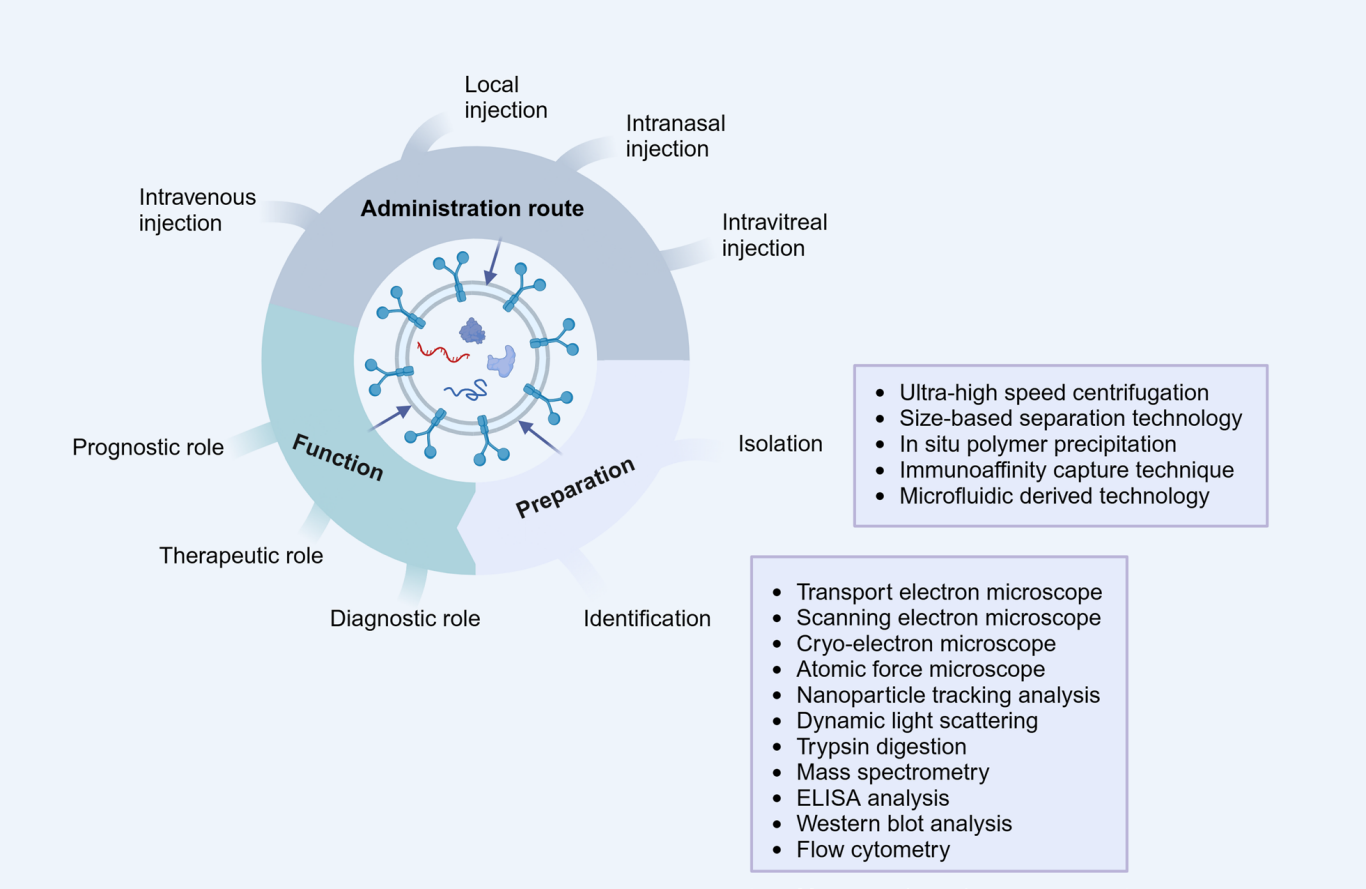
The preparation, functions, and administration routes of exosomes

Depending on the source and size of the exosomes, different techniques can be used to isolate exosomes from body fluids or cell cultures. To date, five exosome isolation techniques have been reported, including ultrahigh-speed centrifugation, size-based isolation, in situ polymer precipitation, immunoaffinity capture, and microfluidic derivations [20] (Fig. 1). Among these, ultrahigh-speed centrifugation is the most commonly used method of exosome isolation [21]. There are multiple techniques available to extract exosomes. However, these technologies have the drawbacks such as low extraction efficiency and poor purity. Therefore, there is an urgent need to further study the extraction techniques to optimize the large-scale production of exosomes for their clinical applications.

### Identification

A variety of techniques have been used to better understand the composition of exosomes. Based on the morphological features, particle size, and surface markers of exosomes, methods for exosome identification include transmission electron microscopy, scanning electron microscopy, cryo-electron microscopy, atomic force microscopy, nanoparticle tracking analysis, dynamic light scattering, trypsin digestion, mass spectrometry, enzyme-linked immunosorbent assay (ELISA), and marker protein expression assays such as Western blot analysis and flow cytometry [22–24] (Fig. 1).

## Functions of exosomes

### Diagnostic role

The natural structure and unique functions of exosomes give them great potential as carriers of natural drugs or genes and high diagnostic potential in immunotherapy, vaccination trials, and regenerative medicine, but the development of efficient and reliable isolation methods is necessary to fully exploit their potential [25]. Exosomes hold promise as diagnostic biomarkers for cardiovascular disease, Parkinson's disease, other neurodegenerative diseases (e.g., Alzheimer's disease), and cancer [26]. In current clinical trials, the use of exosomes as diagnostic biomarkers is based on their role in intercellular communication and disease progression, as well as the loading of related cargoes such as miRNAs, various noncoding RNAs, mitochondrial RNAs, and surface proteins [27–29].

### Therapeutic role

Exosome treatment strategies can be categorized as direct, indirect, or alternative therapies. Direct therapies refer to the use of exosomes as therapeutic agents. Direct therapies take advantage of the ability of exosomes to transfer proteins and nucleic acids between cells and use exosomes as drug carriers to treat disease or injury [26]. Indirect methods refer to the use of exosomes as biomarkers. Exosomes are present in the bloodstream and in all body fluids and exhibit high stability over a wide range of temperatures when isolated [30]. These properties reduce the cost of storage and transport and increase the clinical value of exosomes as biomarkers. Alternative approaches include the elimination of disease-promoting exosomes [31]. Exosomes can be used to stop disease progression by ablating the presence of exosomes that contain harmful disease-associated cargoes, such as viral miRNAs, proteins, and immunosuppressive factors, which promote tumorigenesis, tumour growth, tumour metastasis, and tumour drug resistance [31–34].

### Prognostic role

Tumour-derived exosome cargo matches the genetic content of parental tumour cells [35]. Moreover, exosomes are stable in the bloodstream and protect their cargo from degradation [36]. Therefore, exosomes are increasingly recognized as novel biomarkers for the prognostic evaluation of patients with cancer. Exosomes components, such as the exosome membrane bound proteins New York esophageal squamous cell carcinoma 1 (NY-ESO-1), human alkaline phosphatase (PLAP), epidermal growth factor receptor (EGFR), antibody to apoptosis inducible factor 6 interacting protein (AlIX) and epithelial cell adhesion molecule (EpCAM), can be used as non-invasive prognostic biomarkers of lung cancer [37]. Serum exosomal miR-10b and miR-21 are independent prognostic factors of early disease-free survival in patients with hepatocellular liver cancer [38]. KRAS mutations in circulating exosomal DNA may serve as a prognostically relevant biomarker for patients with early-stage pancreatic cancer [39]. Detection of androgen receptor splice variant 7 (AR-V7) in plasma-derived exosomal RNA significantly predicts resistance to hormone therapy in patients with metastatic prostate cancer, making it a potentially prognostically relevant biomarker [40]. Plasma circulating exosomes carrying PSMA3 and lncPMSA3-AS1 in patients with multiple myeloma are significantly associated with progression-free survival and overall survival [41]. Numerous studies have shown that the prospect of using exosomes for prognostic assessment is intriguing, but their use as prognostic biomarkers of cancer still faces significant challenges. Therefore, further studies are urgently needed to identify reliable exosome biomarkers in large samples for clinical applications. Although exosomes have not been used for the prognostic evaluation of SCI, this may be a new research direction in the future.

## Administration routes of exosomes

### Intravenous injection

Intravenous injection of exosomes is a commonly used methods of tissue repair. Huang et al. demonstrated that intravenous injection of epidural adipose tissue-derived mesenchymal stromal cell-derived extracellular vesicles (MSCs-EVs) could improve the repair effect of SCI by inhibiting the activation of the NLRP3 inflammasome and reducing the expression of inflammatory factors [42]. Other researchers showed that intravenous injection of exosomes secreted by miR-29b-modified bone marrow mesenchymal stromal cells could repair SCI in rats [17]. Overall, intravenous injection is one of the most commonly used methods in experiments because there are no barriers to absorption or first-pass metabolism, and this route has the highest bioavailability among the various delivery methods, allowing long-term therapy.

### Local injection

Local injection of gingival mesenchymal stromal cell-derived exosomes (GMSCs-Exos) significantly reduced periodontal bone resorption and the number of tartrate-resistant acid phosphatase (TRAP)-positive osteoclasts, and these effects were further enhanced by pretreating GMSCs with tumour necrosis factor α (TNF-α) [43]. Local injection of miR-6924-5p-rich exosomes derived from genetically modified scleraxis-overexpressing PDGFRα (+) BMMSCs significantly reduced osteoclast formation and then improved the healing strength of tendons and bone [44]. Local injections are difficult because multiple repeated administrations are required, although the drug concentration is high.

### Intranasal injection

Neuroinflammation-induced migration of mesenchymal stromal cell-derived exosomes (MSCs-Exos) has been shown to have a high affinity for neurons in the lesion after intranasal administration. Intranasal administration of exosomes resulted in significant motor improvements, sensory recovery, and faster recovery of urinary reflexes. Functional recovery was associated with biological changes, such as decreased neuroinflammation, increased axonal regeneration, angiogenesis promotion, and improved electrophysiological signals [45]. Depression-like behaviour was ameliorated in mildly stressed mice after intranasal injection of a miR-139-5p antagonist, suggesting that increased levels of exosomal miR-139-5p may mediate the stress-induced depression-like behaviour in mice [46]. However, there have been few studies on the transnasal administration of exosomes, and many studies are needed to evaluate the efficacy.

### Intravitreal injection

Intravitreal injection of exosomes is another important method for tissue repair and is mainly used to treat retinal disease. Mead et al. [47] have reported that intravitreal injection of BMSCs-Exos promoted the survival of retinal ganglion cells through miRNA-dependent mechanisms. Wang et al. [48] have demonstrated that intravitreal injection of an exosome-associated adeno-associated viral vector enhanced retinoschisin 1 gene transduction in the mouse retina. Moisseiev and colleagues [49] have found that intravitreal administration of MSCs-Exos protected against retinal ischaemia. Intravitreal injection of exosomes has rarely been reported in the literature, but it is a safe, reliable, and effective method of exosomes administration to treat ophthalmic disorders.

Overall, the routes of administration of exosomes include intravenous, local, and intranasal administration and intravitreal injection (Fig. 1). However, there have been few comparative studies on the advantages and disadvantages of these methods, which need to be further studied by researchers in the future.

## Pathological mechanisms of SCI

### Pathological process of SCI

SCI is pathophysiologically divided into primary and secondary injuries [1]. Primary injury occurs when the spine is suddenly injured, resulting in fractures and vertebral displacements characterized by bone fragments and ruptures of the spinal cord ligaments, including destruction of the nerve parenchyma, axonal network destruction, haemorrhage, and disruption of glial membranes [50]. Secondary injury is a cascade of responses triggered by the primary injury, and it is a progressive disease characterized by increased production of proinflammatory cytokines, reactive oxygen species, oxidative damage, and excitatory amino acids (e.g., glutamate), loss of ion homeostasis, mitochondrial dysfunction, and cell death [51].

**Fig. 2:**
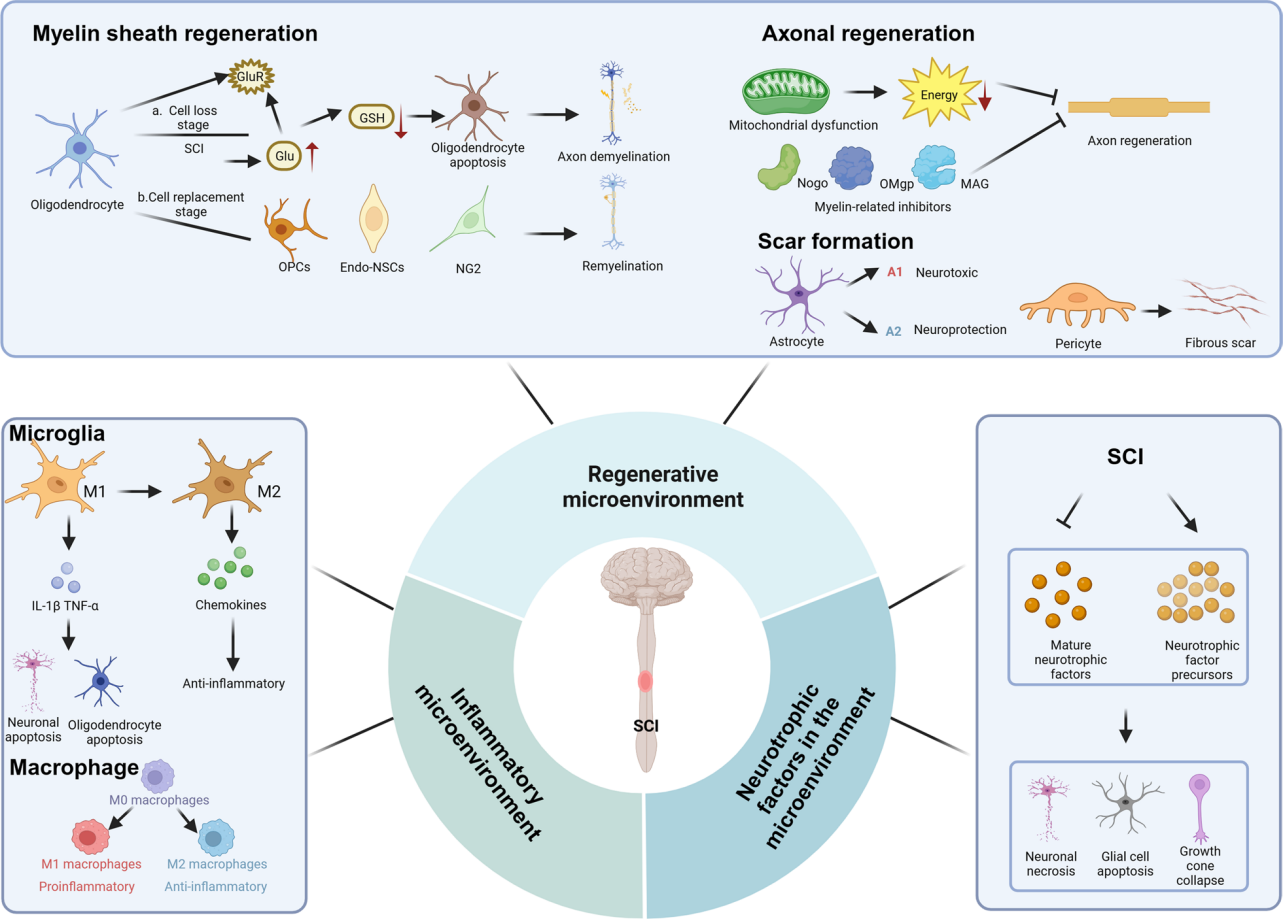
Pathological microenvironment of SCI

SCI can be temporally divided into the acute (< 48 h), subacute (48 h to 14 days), intermediate (14 days to 6 months), and chronic (> 6 months) phases [52]. Based on previous studies, we classified the factors that affect SCI recovery as the inflammatory microenvironment, neurotrophic factors in the microenvironment, and the regenerative microenvironment (Fig. 2).

### Inflammatory microenvironment

Microglia play an important role in the activation and regulation of neuroinflammation after SCI. Microglia can be classified as M1 and M2 types based on their functional role in the inflammatory response, and M1 cells are considered proinflammatory, while M2 cells are considered anti-inflammatory [53–55]. Therefore, finding factors that promote the polarization of microglia towards the M2 phenotype is of great importance for SCI. Furthermore, activated microglia release proinflammatory cytokines, proteases, or other cytotoxic factors, which lead to secondary SCI [56]. Meanwhile, activated microglia may also play a beneficial role by inhibiting lesion expansion, clearing debris, producing anti-inflammatory factors, and attracting numerous immune cells to penetrate the damaged blood-spinal cord barrier (BSCB) [57–59].

Macrophages are derived from blood monocytes and form an innate immune defence together with microglia. David et al. reported that bone marrow-derived macrophages invade lesion centres, phagocytose apoptotic and necrotic cells, and remove tissue debris after SCI [56]. However, nuclear factor kappa β (NF-κβ), signal transducer and activators of transcription 1 (STAT1), and interferon regulatory factor 5 (IRF-5) promote macrophage differentiation into the M1 subtypes, which is proinflammatory. In addition, reactive oxygen species (ROS), reactive nitrogen species (RNS), and lipid peroxidation mediate M1 macrophage activation and the conversion from the M2 phenotype to the M1 phenotype [60, 61]. Furthermore, macrophages also release inflammatory cytokines that exacerbate secondary SCI [57, 59]. Therefore, macrophages are a double-edged sword in the repair of SCI.

### Trophic factors in the microenvironment

Trophic factors mainly include brain-derived neurotrophic factor, glial cell line-derived neurotrophic factor, nerve growth factors, hepatocyte growth factor (HGF), insulin-like growth factor-1 (IGF-1), fibroblast growth factor and neurotrophic factor 3. Neurotrophic factors are crucial for the repair of SCI because promote axonal regeneration. Insufficient secretion of mature neurotrophic factors and increased expression of precursors after SCI can lead to apoptosis and necrosis in neurons and result in the collapse of growth cones, which are important factors that limiting nerve regeneration.

### Regenerative microenvironment

#### Myelin sheath regeneration

The myelin sheath refers to a layer of membrane wrapped around the axons of nerve cells, which is composed of Schwann cells. The myelin sheath has the following functions: (1) to avoid interference between axons and surrounding tissues; (2) accelerate the transmission of action potentials through “skip conduction”; and (3) guide axon regeneration after axon damage. Thus, myelin regeneration is one of the important links in the recovery of neural function after SCI.

In the acute phase of SCI, the number of oligodendrocytes that die and the integrity of myelin are disrupted [62, 63]. In the subacute phase of SCI, oligodendrocyte progenitor cells (OPCs) are activated and begin to differentiate into new oligodendrocytes. In addition, some OPCs may differentiate into Schwann cells. Endothelial cells are activated, and some of these cells differentiate into oligodendrocytes. In the chronic phase of SCI, the new-born oligodendrocytes form myelin around the preserved or regenerated axons [2].

#### Axonal regeneration

In the acute phase of SCI, axonal degeneration is enhanced by oligodendrocyte death and myelin degradation [2]. In the subacute phase of SCI, axons are demyelinated and remodelled [64]. In the chronic phase of SCI, axonal necrosis occurs and then the number of axons decreases and reaches a minimum [65, 66].

Various harmful factors can influence axonal regeneration after SCI. First, microtubules become unstable, disorganized, and unevenly distributed, leading to an low supply of growth cones and impaired axonal contraction [67]. Second, mitochondrial dysfunction can lead to a lack of energy supply to neurons and vascular endothelial cells, ultimately hindering axonal regeneration [68]. Thus, axonal regeneration after SCI requires the restoration of mitochondrial function to ensure sufficient energy supply [69]. Li et al. [70] found that fgf13-induced microtubule stabilization enhanced mitochondrial axonal transport. Third, a membrane-associated protein belonging to the reticulin family, oligodendrocyte myelin glycoprotein, and myelin-associated glycoprotein have been identified as myelin-associated inhibitors that can collapse axon growth cones and inhibit neurite growth [71–75]. Therefore, it is crucial to conduct in-depth research on the relevant mechanisms that prevent axonal regeneration.

#### Scar formation

Scarring after SCI proceeds as follows: In the acute phase of SCI, astrocytes polarize towards the A1 and A2 phenotypes, and pericytes migrate from blood vessels towards the centre of the injury. In the subacute phase of SCI, the scar is formed by natural astrocytes, OPCs, and astrocytes derived from neural stem cells (NSCs). In addition, pericytes derived from fibroblasts close the scar. In the chronic phase of SCI, the scar is stabilized, limiting inflammation and inhibiting axonal regeneration [2].

Astrocytes and pericytes are the main cells involved in scar formation after SCI. Astrocytes are an integral part of the CNS and play an important role in normal neuronal development, synapse formation, neuronal circuit function, and action potential propagation [76–78]. Astrocytes have been classified as A1 and A2 subtypes. A1 astrocytes have upregulated the expression of complement cascade genes that disrupt synapses and damage the nervous system, while A2 astrocytes have upregulated the expression of several neurotrophic factors that may be neuroprotective [79]. Hara et al. [80] reported that type I collagen was highly expressed in the spinal cord during the scar-forming phase and induced astrocytic scar formation via the integrin–N-cadherin pathway. Moreover, pericytes have long projections that surround the vessel wall and are involved in regulating blood flow and the development, maturation, and remodelling of vessels [81–83]. Subsequently, scholars have shown that inhibiting fibrous scar formation caused by pericytes is beneficial for axonal regeneration [84–86]. In summary, in-depth research on scar formation after SCI related to astrocytes and pericytes is crucial for improving neurological function recovery after SCI.

## Exosome-mediated repair of SCI

### Neural stem cell-derived exosomes (NSCs-Exos)

NSCs can differentiate into neurons, oligodendrocytes, and astrocytes and have the ability to repair SCI [87–89]. However, NSC transplantation has many disadvantages that are difficult to overcome. First, it is difficult to pass the BSCB due to a large cell size; second, large cell size blocks distal blood vessels leading to local tumour formation, and third, exogenous NSCs have low survival rates in vivo. Therefore, these factors lead to limited repair of SCI by NSCs [90, 91]. Subsequently, NSCs-Exos, which are derived from NSCs, were attempted to be applied to the repair of SCI and showed encouraging results [92, 93]. Ma et al. claimed that NSCs-Exos promoted neural regeneration by regulating miR-219a-2-3p to repair the SCI [94]. Chen et al. reported that NSCs-Exos promoted neuronal morphology remodelling and improved nerve function in the lower limbs by regulating the PTEN/AKT pathway [95]. Zhong et al. [96] showed that NSCs-Exos were able to transfer vascular endothelial growth factor A (VEGF-A) into spinal microvascular endothelial cells and improve SCI repair through the proangiogenic effects of VEGF-A. Moreover, Zhang et al. [97] showed that NSCs-Exos suppressed neuronal cell apoptosis by activating autophagy via the miR-374-5p/STK-4 axis in SCI. Therefore, NSCs-Exos are a novel and promising substance for the repair of SCI.

### Schwann cell-derived exosomes (SCs-Exos)

SCs are glial cells associated with peripheral nerves that repair injuries to peripheral and central nerves. SCs, which are an important component of the myelin sheaths, have been shown to play an active role in nerve repair [98, 99]. Nevertheless, the therapeutic efficacy of SC transplantation in promoting functional recovery after SCI is limited due to the poor survival rate of SCs after transplantation and the difficulty of overcoming the blood–brain barrier (BBB) [100, 101]. Then, SCs-Exos have been shown to facilitate axonal regeneration and inhibit inflammatory responses [102, 103]. Scholars found that SCs-Exos could promote angiogenesis through the upregulation of integrin-β1, inhibit neuronal apoptosis through the modulation of the EGFR/Akt/mTOR pathway, and affect the anti-inflammatory response through the modulation of the SOCS3/STAT3 pathway, which improved the recovery effect of motor function after SCI [104–107]. Thus, SCs-Exos are an alternative option for repairing SCI by promoting myelin regeneration.

### Mesenchymal stromal cell-derived exosomes (MSCs-Exos)

**Fig. 3:**
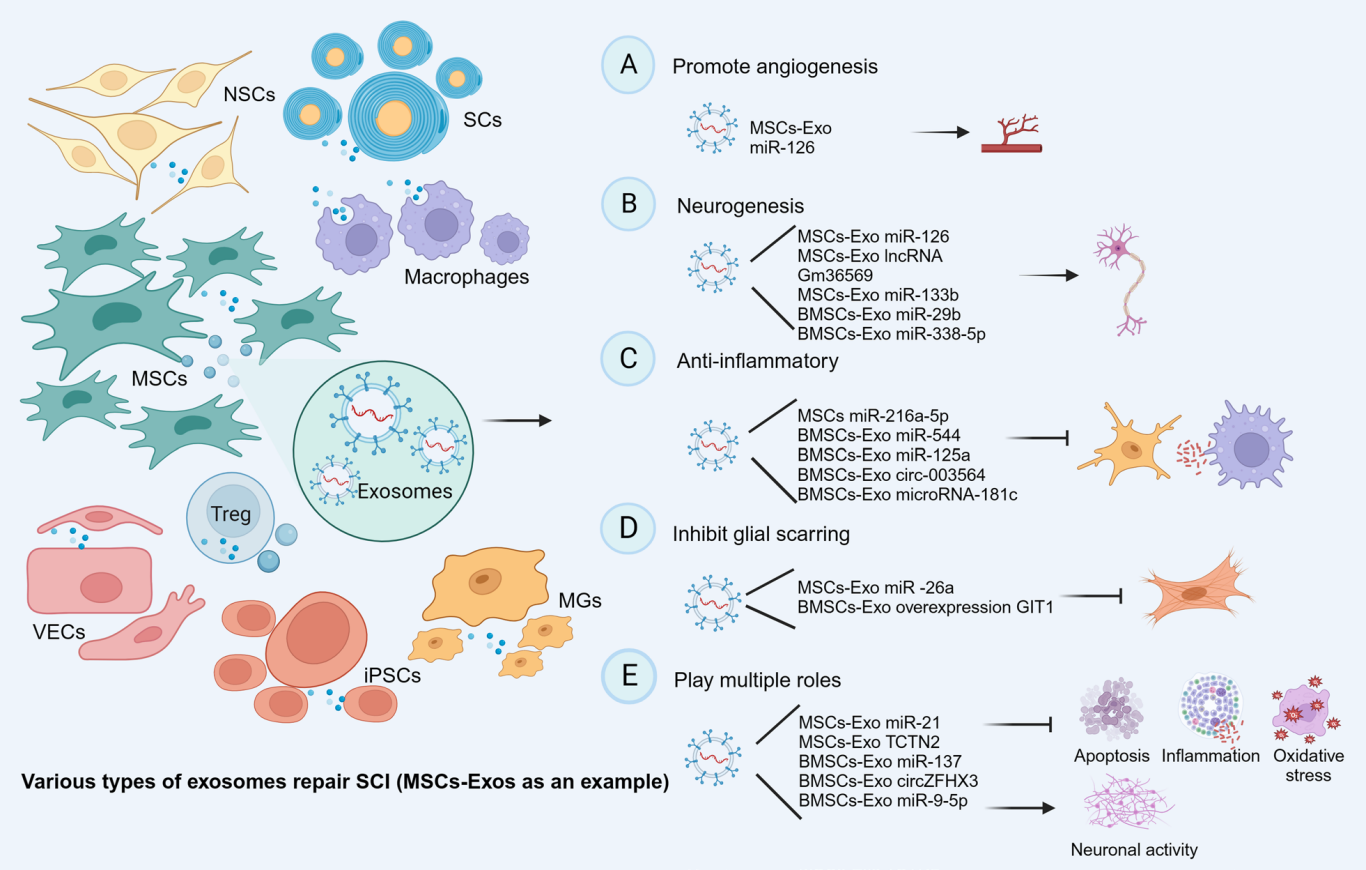
Various types of exosomes repair SCI (MSCs-Exos as an example)

MSCs are pluripotent stem cells that can be derived from various cells, such as bone marrow, adipose tissue, and umbilical cord. In particular, MSCs-Exos can maintain the integrity of the BSCB and promote angiogenesis, proliferation and antioxidant effects, as well as immunomodulatory, anti-inflammatory, and antiapoptotic effects [108]. MSCs-Exos, which are derived from MSCs, are enriched in important MSC substances [109]. Accordingly, various MSCs-Exos, such as HUCMSCs-Exos, BMSCs-Exos and engineered MSCs-Exos, promote nerve regeneration, facilitate angiogenesis, inhibit the inflammatory response, suppress oxidative stress, block apoptosis, and reduce glial scar formation, thus working to repair SCI [92, 110–113]. Furthermore, a multiple signalling pathways associated with SCI repair by MSCs-Exos have been described, including PTEN/AKT, PTEN-AKT-mTOR, TLR4/NF-κβ, TLR4/MyD88/NF-κβ, ROS/MAPK/NF-κβ/P65, TIMP2/MMP, miR-21/PTEN/PDCD4, and miR-329-3p/IGF1R axes [110, 114–119]. Indeed, one type of MSCs-Exos can repair SCI through multiple pathways; for example, engineered BMSCs-Exos can simultaneously inhibit neuronal apoptosis and promote vascularization (Fig. 3). Thus, MSCs-Exos may provide new insights for repairing SCI.

### Astrocyte-derived exosomes (AS-Exos)

Astrocytes are the most common glial cells in the CNS and play a role in supporting the BBB, controlling ionic homeostasis, and regulating neuronal function in the CNS [120, 121]. Recently, scholars have found that AS-Exos play an important role in CNS injury. Under physiological conditions, AS-Exos contains proteins and miRNAs that promote neuronal survival and axonal growth [122, 123]. However, under pathological conditions, AS-Exos may promote neuronal apoptosis [124]. Long et al. [125] reported that AS-Exos could promote microglial polarization towards the M2 phenotype by inhibiting ERK and NF-κβ p65 phosphorylation, thereby attenuating neurological deficits due to traumatic brain injury (TBI). Zhang et al. [95] suggested that AS-Exos could activate the Nrf2 signalling pathway to inhibit neuronal oxidative stress and neuronal apoptosis, thus relieving TBI. Chen et al. [126] have found that AS-Exos carrying GJA1-20k can decrease Cx43 phosphorylation, protect mitochondrial function, and decrease the rate of neuronal apoptosis, thus promoting neuronal recovery. Studies have shown that AS-Exos could reduce cerebral ischaemia/reperfusion-induced neurological injury by targeting TLR7 to downregulate the NF-κβ/MAPK pathway [127]. Although studies have used AS-Exos to treat TBI, few have used them to treat SCI. However, both TBI and SCI are CNS injuries, and there are similarities in the injury mechanisms and treatment options. We believe that more and more research will be done in the future on AS-Exos-mediated repair of SCI and that AS-Exos are a very promising factors for repairing SCI.

### Platelet-rich plasma-derived exosomes (PRP-Exos)

To date, few studies have focused on PRP-Exos for the repair of SCI, but numerous reports have focused on platelet-rich plasma extracellular vesicles (PRP-EVs) for the treatment of SCI [128]. PRP-Exos and PRP-EVs are PRP derivatives that are similar in their major components and differ only in diameter. Thus, in this section, we reviewed the role of PRP-EVs in repairing SCI. PRP-EVs are enriched with a variety of important biomolecules, including growth factors, cytokines, chemokines, lipids, and nucleic acids, which are able to influence coagulation, inflammatory response, and neovascularization; therefore, PRP-EVs have promising applications in the field of tissue repair and regeneration [129–131]. In recent years, many scholars have conducted preclinical studies of PRP-EVs used in myocardial ischaemia [132, 133], cerebral ischaemia [134], atherosclerosis [135, 136], vascular damage [137], haemorrhagic shock [138], wound healing [139, 140], acute lung injury [141], necrosis of the femoral head [142], and osteoarthritis [143]. However, there have been few studies related to PRP-EVs in the treatment of SCI. Therefore, this is a direction for future research. We believe that the study of PRP-Exos and PRP-EVs in SCI repair is in its early stage, and a large number of in-depth studies are still needed to evaluate their role in repairing SCI.

### Macrophage-derived exosomes (M-Exos)

Macrophages are an important kind of immune cell and are classified into two subtypes: M1 and M2 [144]. M1 macrophages are able to secrete substances that promote the inflammatory response, which leads to the aggravation of SCI; however, M2 macrophages inhibit the inflammatory response and promote angiogenesis, which helps to repair SCI [145–148]. Therefore, the opposite roles of M1 and M2 macrophages in the SCI microenvironment have received increasing attention. Huang et al. [149] found that M2 macrophage-derived exosomes (M2-Exos) were able to activate the HIF-1/VEGF signalling pathway to promote neovascularization and thus play a role in repairing SCI. Peng et al. [150] noted that M2-Exos induced the transformation of M1 macrophages into M2 macrophages by regulating the miR-23a-3p/PTEN/PI3K/AKT signalling axis, thereby improving the local microenvironment after SCI. Moreover, Zhang et al. also reported that peripheral macrophage-derived exosomes (PM-Exos) promoted repair after SCI by inducing local polarization of anti-inflammatory microglia by inhibiting the PI3K/AKT/mTOR pathway [151]. Thus, M-Exos is critical in SCI repair.

### Vascular endothelial cell-derived exosomes (VECs-Exos)

Ge et al. [152] found that VECs-Exos could enhance M2 microglia/macrophage polarization and improve functional recovery by delivering USP13 and concluded that VECs-Exos were promising agents for the repair of SCI. Unfortunately, there have been very few studies on the repair of SCI by VECs-Exos, and more and more insightful experimental research is necessary.

### Induced pluripotent stem cell-derived exosomes (iPSCs-Exos)

Li et al. [153] reported that iPSCs-Exos carrying miR-199b-5p could treat SCI confirmed that miR-199b-5p induced macrophage polarization and SCI recovery by regulating the HGF and phosphoinositide 3-kinase (PI3K) signalling pathways and concluded that the miR-199b-5p-bearing iPSCs-Exos might become an effective method to treat SCI. At present, few studies have been conducted on the treatment of SCI by iPSCs-Exos, which need to be further studied in the future.

### Microglia-derived exosomes (MG-Exos)

Microglia are a type of immune cell found in the CNS that protects neuronal cells. Li et al. [154] found that MG-Exos inhibited neuronal apoptosis and promoted axon growth by regulating the p53/p21/CDK1 signalling pathway, which had neuroprotective effects. In addition, Peng et al. [155] claimed that MG-Exos could improve spinal cord functional recovery after injury by inhibiting oxidative stress and promoting the survival and function of endothelial cells by activating the Keap1/Nrf2/HO-1 signalling pathway. Moreover, Huang et al. [156] found that the increase in miR-124-3p in MG-Exos following TBI could inhibit neuronal inflammation and contribute to neurite outgrowth after being transferred into neurons. Thus, MG-Exos are also an optional, promising and important substance for the treatment of SCI.

### Regulatory T cell-derived exosomes (Treg-Exos)

Treg cells are present within all tissues and supress excessive immune activation to curb autoimmunity and maintain immune homeostasis [157]. Xiong and colleagues [158] found that Treg cell-derived exosomal miR-709 attenuated microglial pyroptosis and promoted motor function recovery after SCI. However, little is known about the exact mechanism by which Treg-Exos promotes the recovery of behavioural function after SCI in mice, which needs to be further explored in future studies.

Various exosomes and their functions in the treatment of SCI are summarized in Table 1.

**Table 1:** Mechanisms of various exosomes to repair SCI

Researchers	Exosomes	Signalling pathways/ cytokines	Effect
Tan et al. [94]	NSCs-Exos	miR-219a-2-3p	(1) Inhibit neuroinflammation (2) Inhibit cell apoptosis (3) Promote nerve regeneration
Chen et al. [95]	NSCs-Exos	PTEN/AKT	(1) Promote neuron morphology (2) Improve hind limb motor behavior
Zhong et al. [96]	NSCs-Exos	VEGF-A	(1) Promote SCMECs angiogenesis (2) Improve nerve function
Zhang et al. [97]	NSCs-Exos	miR-374-5p/STK-4	(1) Activate autophagy
Huang et al. [104]	SCs-Exos	Integrin-β	(1) Promote the proliferation, migration and tubular formation of endothelial cells (2) Facilitate angiogenesis
Ren et al. [105]	SCs-Exos	SOCS3/STAT3	(1) Anti-inflammatory (2) Reduce neuronal apoptosis
Pan et al. [106]	SCs-Exos	EGFR/Akt/mTOR	(1) Increase autophagy (2) Decrease apoptosis (3) Induce axonal protection
Pan et al. [107]	SCs-Exos	NF-κβ/PI3K	(1) Increase the expression of TLR2 on astrocytes (2) Decrease the deposition of CSPGs
Yuan et al. [110]	Pericytes exosomes	PTEN/AKT	(1) Promote blood flow (2) Improve endothelial function (3) Protect the BBB (4) Alleviate apoptosis
Zhou et al. [112]	HPMSCs-Exos	NPCs	(1) Enhance neurogenesis
Sheng et al. [192]	BMSCs-Exos	Regulate MARCO expression in macrophages	(1) Promote macrophages to phagocytose myelin debris (2) Promote axon regeneration
Li et al. [193]	BMSCs-Exos	NGF-overexpressing	(1) Promote NSCs differentiation (2) Promote axon regeneration
Wang et al. [114]	MSCs-Exos	NF-κβ p65	(1) Reduction of A1 astrocytes (2) Anti-inflammatory (3) Neuroprotective
Fan et al. [115]	BMSCs-Exos	TLR4/MyD88/NF-κβ	(1) Inhibit apoptosis and inflammation (2) Promote the recovery of motor function
Liu et al. [92]	BMSCs-Exos	Inhibit the activation of A1 neurotoxic reactive astrocytes	(1) Promote the formation of blood vessels (2) Inhibit neuronal cell apoptosis (3) Anti-inflammatory (4) Promote axon regeneration
Kang et al. [165]	HUCMSCs-Exos	BCL2/Bax and Wnt/β-catenin	(1) Antiapoptotic (2) Anti-inflammatory
Xin et al. [117]	BMSCs-Exos	TIMP2/MMP	(1) Alleviate BSCB destruction (2) Improve functional recovery
Shao et al. [160]	MSCs-Exos	miR-5627-5p/FSP1	(1) Inhibit iron death of neuron cells (2) Improve nerve function
Li et al. [162]	MSCs-Exos	ERK1/2, STAT3, CREB, RhoA	(1) Preserve neurons (2) Enhance axon regeneration
Yu et al. [17]	BMSCs-Exos	NF200, GAP—43 and GFAP	-
Zhang et al. [168]	BMSCs-Exos	PI3K/Akt	(1) Neuroprotective effect
Jiang et al. [53]	MSCs-Exos	TLR4/NF-κβ and PI3K/AKT	(1) Microglia changed from M1 to M2
Chang et al. [194]	BMSCs-Exos	IRF5	(1) Promote the polarization of M2 macrophages
Zhang et al. [195]	BMSCs-Exos	PTEN and NF-κβ	(1) Inhibit inflammation of microglia and spinal cord
Chen et al. [118]	MSCs-Exos	PTEN/AKT/mTOR	(1) Inhibit the formation of glial scar
Luo et al. [196]	BMSCs-Exos	GIT1 overexpression	(1) Inhibit the formation of glial scar
Kang et al. [119]	MSCs-Exos	miR-21/PTEN/PDCD4	(1) Promote functional recovery (2) Inhibit cell death
Liu et al. [172]	MSCs-Exos	miR-329-3p/IGF1R	(1) Alleviates LPS-induced neuronal apoptosis, inflammation, and oxidative stress
Tian et al. [169]	BMSCs-Exos	miR-16-5p/IGF-1	(1) Improve cell viability (2) Inhibition of cell apoptosis, inflammation and oxidative stress
He et al. [197]	BMSCs-Exos	Promote HDAC5 deacetylation and mediated FGF2 expression	(1) Inhibition of LPS-induced apoptosis of PC12 cells (2) Reduce inflammation and endoplasmic reticulum stress
Huang et al. [149]	M2-Exos	HIF-1/VEGF	(1) Promote angiogenesis (2) Improve nerve function recovery
Peng et al. [150]	M2-Exos	miR-23a-3p/PTEN/PI3K/AKT	(1) Regulate the phenotype of macrophages (2) Improve the damaged immune microenvironment
Luo et al. [198]	M2-Exos	Deubiquitinating β-catenin and triggering the expression of genes associated with angiogenesis	(1) Mediate angiogenesis
Zhang et al. [151]	PM-Exos	PI3K/AKT/mTOR	(1) Activation of microglial autophagy (2) Enhance the polarization of anti-inflammatory microglia
Ge et al. [152]	VECs-Exos	USP13	(1) Improve functional recovery (2) Enhance microglia/macrophage towards to M2 polarization
Li et al. [154]	MG-Exos	p53/p21/CDK1	(1) Inhibit neuronal apoptosis (2) Promote axon growth (3) Neuroprotective effect
Peng et al. [155]	MG-Exos	Keap1/Nrf2/HO-1	(1) Promote functional recovery
Xiong et al. [158]	Treg-Exos	NKAP	(1) Reduce microglia pyroptosis

*NSCs-Exos* neural stem cell-derived exosomes, *IGF-1* insulin-like growth factor-1, *SCs-Exos* Schwann cell-derived exosomes, *HPMSCs-Exos* human placental mesenchymal stem cells-derived exosomes, *BMSCs-Exos-EEM* bone marrow mesenchymal stromal cell-derived exosome-educated macrophages, *MSCs-Exos* mesenchymal stromal cell-derived exosomes, *HUCMSCs-Exos* human umbilical cord mesenchymal stem cell-derived exosomes, *M2-Exos* M2 macrophage-derived exosomes, *PM-Exos* peripheral macrophage-derived exosomes, *iPSCs-Exos* induced pluripotent stem cell-derived exosomes, *MG-Exos* microglia-derived exosomes, *NPCs* neuronal progenitor cells, *NGF* nerve growth factor, *HIF-1* hypoxia-inducible factor-1, *VEGF* vascular endothelial growth factor, *SCMECs* spinal cord microvascular endothelial cells, *BSCB* blood spinal cord barrier, *LPS* lipopolysaccharide, *SCI* spinal cord injury, *CSPGs* chondroitin sulphate proteoglycans

## Mechanisms by which exosomes repair SCI

### Promote nerve regeneration

**Fig. 4:**
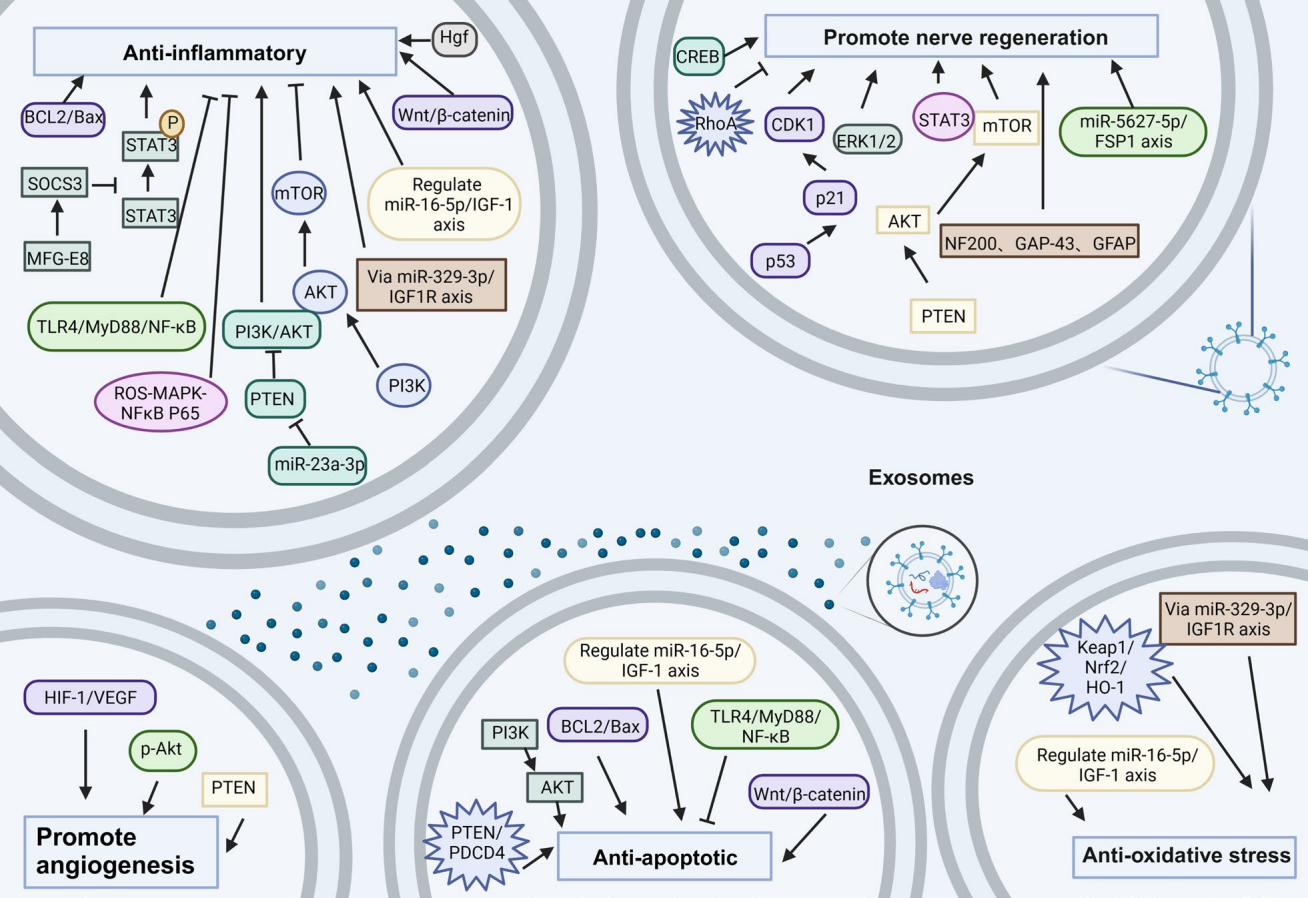
Mechanisms of exosomes repair SCI

Neurons are the basic structural and functional units of the nervous system. SCI can result in neuronal damage [159]. Chen et al. [95] claimed that FTY720-loaded NSCs-Exos therapy could promote neuronal morphology by modulating the PTEN/AKT pathway and improve hindlimb motor function. SCs-Exos and MG-Exos have protect axons through the EGFR/Akt/mTOR and p53/p21/CDK1 pathways, respectively [106, 154]. Moreover, many researchers found that MSCs-Exos could inhibit neuronal cell death and promote axonal regeneration through various pathways, including the miR-5627-5p/FSP1, ERK1/2, and PTEN-AKT-mTOR pathways [118, 160–162]. Thus, promoting axonal regeneration is one of the ways to repair SCI (Fig. 4).

### Promote angiogenesis

Vascularization facilitates nerve regeneration and promotes neurovascular unit recovery after SCI [163]. Yuan et al. [110] found that pericyte exocytosis could promote blood flow, improve vascular endothelial function, and protect the BSCB via the PTEN/AKT signalling pathway, which is beneficial for SCI recovery. Huang et al. [149] showed that M2 macrophage-derived exosomes could promote angiogenesis after SCI through activation of the HIF-1/VEGF pathway. Thus, angiogenesis and improvements in vascular endothelial function promoted by exosomes are one of the ways to repair the SCI (Fig. 4).

### Anti-inflammatory effects

Microglia and macrophages can release cytokines and chemokines after SCI, leading to neuroinflammatory responses [164]. According to the previous studies, exosomes that exert anti-inflammatory effects can be categorized into multiple groups. First, exosomes such as HUCMSCs-Exos can inhibit microglia and macrophage activation through the BCL2/Bax and Wnt/β-catenin signalling pathways [165]. Second, exosomes such as SCs-Exos and M2-Exos inhibit M1 macrophages/microglia polarization and promote M2 polarization via the SOCS3/STAT3 and miR-23a-3p/PTEN/PI3K/AKT pathways [105, 150]. Third, exosomes such as BMSCs-Exos and MSCs-Exos induce differentiation of microglia or macrophages of the M1 subtype toward the M2 type by inhibiting the TLR4/MyD88/NF-κβ and promoting the PI3K/AKT pathways [54, 115]. Fourth, exosomes such as PM-Exos can activate autophagy in microglia and enhance anti-inflammatory effects by inhibiting the PI3K/AKT/mTOR pathway [151]. Thus, exosomes can inhibit neuroinflammatory responses through multiple signalling pathways to repair SCI (Fig. 4).

### Antiapoptotic effects

Apoptosis is a type of programmed cell death that plays an important role in SCI, especially in secondary SCI. It occurs in glial cells, oligodendrocytes, neurons, and vascular endothelial cells and severely affects the recovery of neurologic function [166, 167]. Therefore, blocking apoptosis after SCI is essential and can improve the recovery effect of SCI. Various types of exosomes, such as NSCs-Exos, BMSCs-Exos or HUCMSCs-Exos, have been shown to have antiapoptotic effects after SCI through different signalling pathways, including the TLR4/MyD88/NF-κβ [115], PI3K/Akt [168], BCL2/Bax and Wnt/β-catenin [165], miR-21/PTEN/PDCD4 [119] and miR-16-5p/IGF-1 pathways [169]. Thus, the antiapoptotic effects of exosomes can repair SCI (Fig. 4).

### Antioxidative stress effects

Lipid peroxidation is one of the characteristic features of secondary SCI, which is caused by significantly increased levels of ROS and RNS and eventually leads to cytokinesis, the destruction of proteins and nucleic acids, and apoptosis or necrosis [170, 171]. Thus, inhibiting oxidative stress is necessary for functional recovery after SCI. Numerous types of exosomes, such as MSCs-Exos or MG-Exos, have been confirmed to have antiapoptotic effects after SCI by regulating multiple signalling pathways, such as the miR-329-3p/IGF1R [172], Keap1/Nrf2/HO-1 [155] and miR-16-5p/IGF-1 pathways [169]. Thus, the antioxidative effect of exosomes can repair SCI (Fig. 4).

## Exosomes combined with other methods to repair SCI

### Exosomes combined with hydrogels

**Fig. 5:**
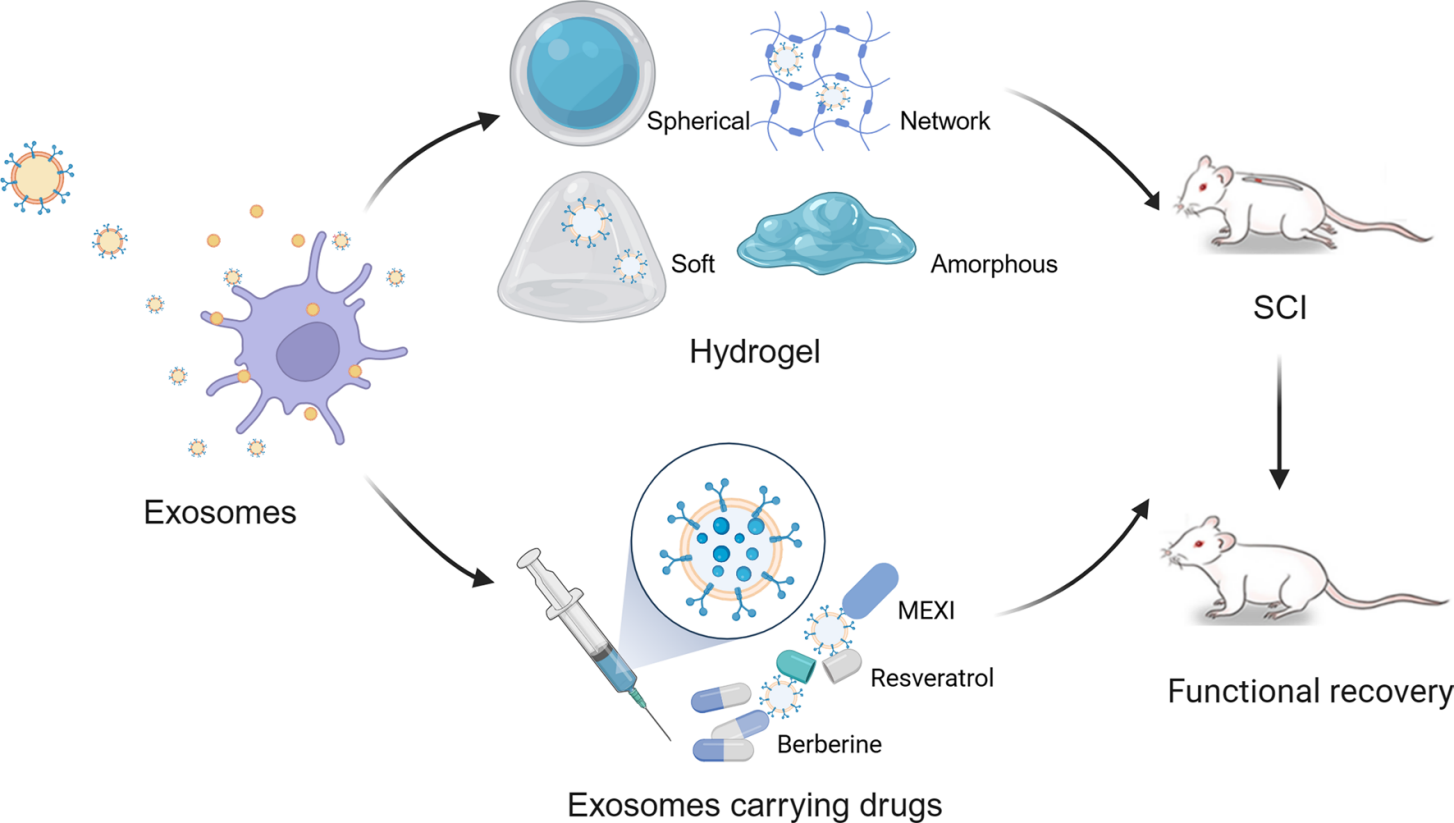
Exosomes combined with other strategies to repair SCI

Although exosomes have been shown to have favourable effects on SCI, scholars have shown that intravenous injection of exosomes has serious drawbacks, including a short half-life and low targetability [173]. Therefore, exploring other types of medical biomaterials to improve the efficacy of exosomes is an urgent problem. Hydrogel is a polymer material with high water content and diverse physical properties. Hydrogels currently used for SCI repair include natural hydrogels such as alginate, agarose, collagen, fibronectin, gelatine, and extracellular matrix, and synthetic hydrogels such as polylactic acid, polylactic acid-glycolic acid, and polyethylene glycol [174]. Researchers showed that exosomes encapsulated by hydrogels offer multiple advantages, including slow rate release of exosomes, the maintenance of exosome bioactivity, and improved exosome targeting, which offer promising prospects for the medical use of exosomes [175]. Many scholars have proven that exosomes combined with hydrogels can provide better anti-inflammatory effects, inhibit glial scar formation, promote axonal regeneration, and promote neovascularization after local injection and long-term release, thus improving neurological function after SCI [174, 176–179] (Fig. 5). Thus, exosomes combined with hydrogels are a promising approach for the repair of SCI.

### Exosomes carrying drugs

Successful delivery of therapeutic agents to target cells and tissues is limited by many factors, including the instability of therapeutic agents in vivo, the isolation of target tissues and the BSCB, and drug efflux systems, among which, the BSCB is one of the major obstacles to SCI repair [173]. However, exosomes show great potential for application as drug carriers due to their relatively small molecular structure, natural molecular transport properties, good biocompatibility, and ability to penetrate BSCB [173, 180]. As a result, researchers have modified exosomes to use them as therapeutic drug carriers. Recently, Zeng et al. [181] identified an azide-modified Ile-Lys-Val-Ala-Val peptide conjugated onto M2-Exos that provided strong evidence of SCI recovery, including inhibiting inflammation, promoting neuronal differentiation of NSCs, and targeting the injured site of the spinal cord after tail vein injection. In addition, Gao et al. [182] found that another combination agent in which berberine was attached to M2-Exos could repair SCI through anti-inflammatory and antiapoptotic effects and by inducing the polarization of M1-type macrophages to the M2 type. Moreover, Yue et al. [183] indicated that exosomes could enhance the solubility of resveratrol and enhance penetration of the drug through the BBB, thereby increasing its concentration in the CNS. Thus, as drug carriers, exosomes have many advantages. They maintain drug stability in the body, improve drug solubility, mediate drug targeting, and facilitate drug crossing of the BBB and BSCB (Fig. 5). Consequently, exosomes will be a promising drug delivery system for the repair of SCI.

## Challenges in the use of exosomes to repair SCI

### Low amounts

**Fig. 6:**
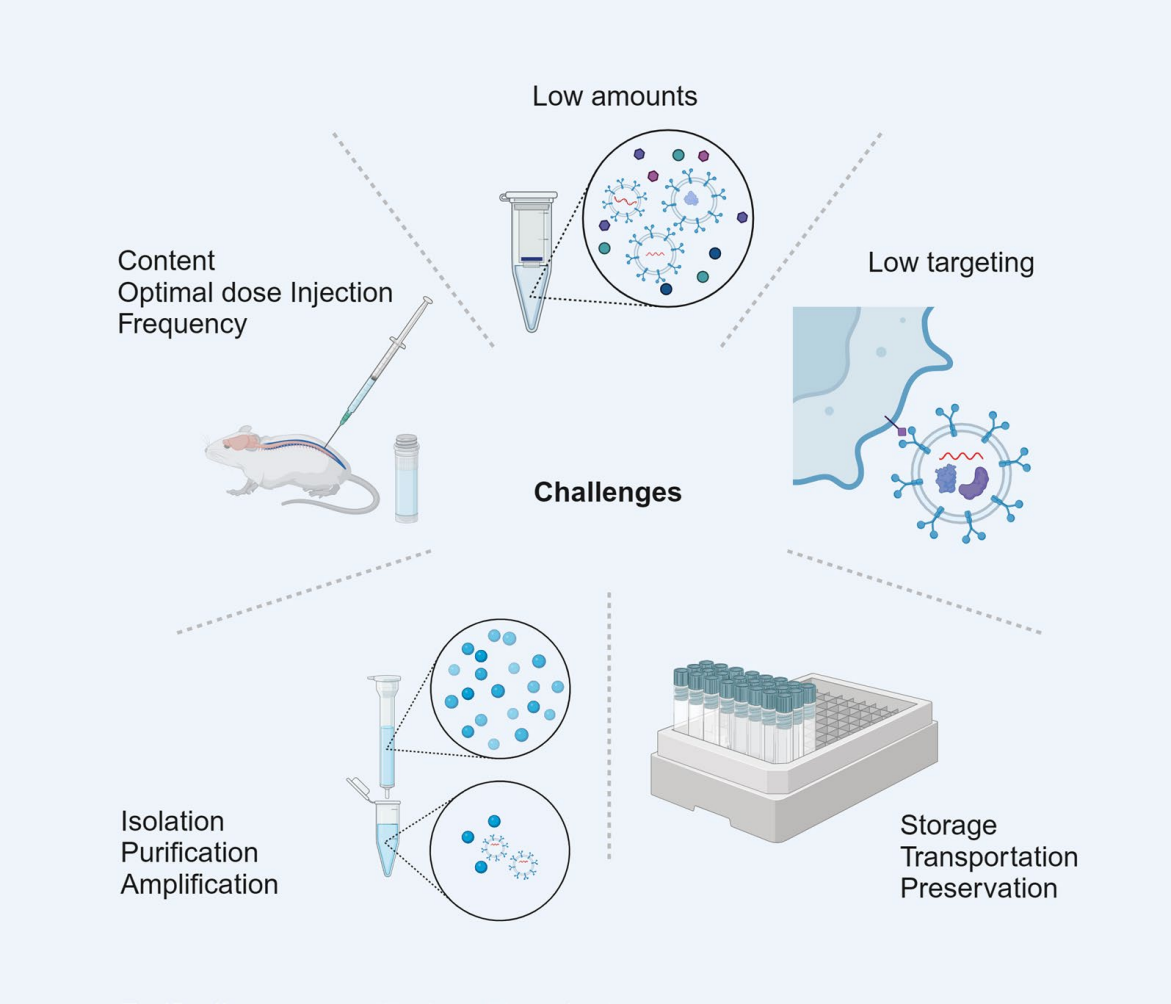
Challenges in the use of exosomes to repair SCI

Currently, various methods of exosome acquisition are available. However, no standardized consensus on exosome extraction methods has been established. Accordingly, exosomes extracted by different methods showed significant differences in protein and RNA levels [184]. Furthermore, even if exosomes are extracted, they are present in very low amounts [185–187]. To date, ultracentrifugation is the most commonly used method of exosome isolation; however, the exosomes acquired by ultracentrifugation may contain other impurities, such as extracellular vesicles with diameters close to those of the exosomes [188] (Fig. 6).

### Low targeting

Scholars attribute the low targeting of exosomes to the fact that intravenously injected exosomes accumulate in the kidneys, liver, and spleen and are rapidly eliminated through glomerular filtration, biliary excretion, and phagocytosis by the reticuloendothelial system, which ultimately leads to poor therapeutic efficacy [173, 189, 190] (Fig. 6).

### Other challenges

Despite the favourable results of exosomes in preclinical studies of SCI, using exosomes to repair SCI in the clinic practice still faces challenges. First, clarifying the conditions for the safe storage, transport, and preservation of exosomes is fundamental to the development of exosome therapy. Second, clarifying the dose, frequency, duration, and method of administration of exosomes for SCI repair is an important measure to ensure therapeutic efficacy [180, 191] (Fig. 6).

## Conclusion

SCI repair remains a challenge for the medical field. Exosomes derived from various cells are important intercellular communication substances that can mediate SCI repair by inhibiting inflammatory responses, promoting neurovascular regeneration, inhibiting scarring, resisting oxidative stress, and reducing cell apoptosis, which affect SCI repair by regulating multiple signalling pathways, including the PTEN-AKT/mTOR, EGFR/Akt/mTOR, p53/p21/CDK1, ERK1/2, HIF-1/VEGF, SOCS3/STAT3, TLR4/MyD88/NF-κβ, ROS/MAPK/NF-κβ P65, BCL2/Bax, Wnt/β-catenin, PI3K/AKT/mTOR, HGF, PTEN/PDCD4 and Keap1/Nrf2/HO-1 pathways. Moreover, engineered exosomes, exosome-carried drugs, and exosome-combined hydrogels are important ways to enhance the repair of SCI and are hot research topics. The use of exosomes for SCI repair also faces disadvantages such as low targeting and a short half-life, but we believe that the existing research results provide sufficient evidence for preclinical studies and the use of exosomes to treat SCI. In summary, exosomes are a promising therapeutic strategy for SCI.

## Data Availability

The data that support the findings of this study are available from the corresponding author upon reasonable request.

## Abbreviations

SCI: Spinal cord injury
CNS: Central nervous system
NSCs-Exos: Neural stem cell-derived exosomes
SCs-Exos: Schwann cell-derived exosomes
MSCs-Exos: Mesenchymal stromal cell-derived exosomes
AS-Exos: Astrocyte-derived exosomes
PRP-Exos: Platelet-rich plasma-derived exosomes
M-Exos: Macrophage-derived exosomes
VECs-Exos: Vascular endothelial cell-derived exosomes
iPSCs-Exos: Induced pluripotent stem cell-derived exosomes
MG-Exos: Microglia-derived exosomes
Treg-Exos: Regulatory T cell-derived exosomes
MSCs-EVs: Mesenchymal stromal cell-derived extracellular vesicle
BMSCs-Exos: Bone marrow mesenchymal stromal cell-derived exosomes
GMSCs-Exos: Gingival tissue mesenchymal stromal cell-derived exosomes
HUCMSCs-Exos: Human umbilical cord mesenchymal stromal cell-derived exosomes
TRAP: Tartrate-resistant acid phosphatase
TNF-α: Tumour necrosis factor α
BSCB: Blood-spinal cord barrier
NF-κβ: Nuclear factor kappa β
STAT1: Signal transducer and activators of transcription 1
IRF-5: Interferon regulatory factor 5
ROS: Reactive oxygen species
RNS: Reactive nitrogen species
HGF: Hepatocyte growth factor
IGF-1: Insulin-like growth factor-1
OPCs: Oligodendrocyte progenitor cells
CSPGs: Chondroitin sulphate proteoglycans
VEGF: Vascular endothelial growth factor
BBB: Blood–brain barrier
TBI: Traumatic brain injury
PRP-EVs: Platelet-rich plasma extracellular vesicles
HIF-1: Hypoxia-inducible factor-1
Ber: Berberine
ELISA: Enzyme-linked immunosorbent assay
NY-ESO-1: New York esophageal squamous cell carcinoma 1
PLAP: Human alkaline phosphatase
EGFR: Epidermal growth factor receptor
AlIX: Antibody to apoptosis inducible factor 6 interacting protein
EpCAM: Epithelial cell adhesion molecule
AR-V7: Androgen receptor splice variant 7
